# Secretion-Positive LGI1 Mutations Linked to Lateral Temporal Epilepsy Impair Binding to ADAM22 and ADAM23 Receptors

**DOI:** 10.1371/journal.pgen.1006376

**Published:** 2016-10-19

**Authors:** Emanuela Dazzo, Emanuela Leonardi, Elisa Belluzzi, Sandro Malacrida, Libero Vitiello, Elisa Greggio, Silvio C. E. Tosatto, Carlo Nobile

**Affiliations:** 1 CNR-Neuroscience Institute, Section of Padua, Padova, Italy; 2 Department of Woman and Child’s Health, University of Padua, Padova, Italy; 3 Department of Biology, University of Padua, Padova, Italy; 4 Department of Biomedical Sciences, University of Padua, Padova, Italy; Columbia University Medical Center, UNITED STATES

## Abstract

Autosomal dominant lateral temporal epilepsy (ADTLE) is a focal epilepsy syndrome caused by mutations in the *LGI1* gene, which encodes a secreted protein. Most ADLTE-causing mutations inhibit LGI1 protein secretion, and only a few secretion-positive missense mutations have been reported. Here we describe the effects of four disease-causing nonsynonymous *LGI1* mutations, T380A, R407C, S473L, and R474Q, on protein secretion and extracellular interactions. Expression of LGI1 mutant proteins in cultured cells shows that these mutations do not inhibit protein secretion. This finding likely results from the lack of effects of these mutations on LGI1 protein folding, as suggested by 3D protein modelling. In addition, immunofluorescence and co-immunoprecipitation experiments reveal that all four mutations significantly impair interaction of LGI1 with the ADAM22 and ADAM23 receptors on the cell surface. These results support the existence of a second mechanism, alternative to inhibition of protein secretion, by which ADLTE-causing *LGI1* mutations exert their loss-of-function effect extracellularly, and suggest that interactions of LGI1 with both ADAM22 and ADAM23 play an important role in the molecular mechanisms leading to ADLTE.

## Author Summary

Temporal lobe epilepsy is the most common form of focal epilepsy. It is frequently associated with structural brain abnormalities, but genetic forms caused by mutations in major genes have also been described. Autosomal dominant lateral temporal epilepsy (ADLTE) is a familial condition characterized by focal seizures with prominent auditory symptoms. ADLTE-causing mutations are found in the *LGI1* gene in about 30% of affected families. *LGI1* encodes a protein, LGI1, that is secreted by neurons. Most *LGI1* mutations suppress protein secretion, thereby preventing protein function in the extracellular environment. In this paper, we examine the effects of four *LGI1* mutations and show that they do not inhibit secretion of the LGI1 protein but impair its interaction with the neuronal receptors ADAM22 and ADAM23. In agreement with these findings, a three- dimensional model of the protein predicts that these mutations have no impact on LGI1 structure but instead may affect amino acids that are critical for interactions with ADAM receptors. Our results provide novel evidence for an extracellular mechanism through which mutant LGI1 proteins cause ADLTE and strengthen the importance of LGI1-ADAM22/23 protein complex in the mechanisms underlying ADLTE.

## Introduction

Mutations in the leucine-rich, glioma-inactivated 1 (*LGI1*) gene cause autosomal dominant lateral temporal epilepsy (ADTLE) [1,2], a genetic epilepsy syndrome characterized by focal seizures with prominent auditory or aphasic symptoms, normal magnetic resonance imaging, and usually benign evolution [3–5]. ADLTE is inherited in autosomal dominant fashion with reduced penetrance [6,7], and *LGI1* mutations are found in about 30% of families with this syndrome [7]. To date, more than 30 ADLTE-causing mutations have been detected throughout the protein-coding region of *LGI1*, resulting in either protein truncation or single amino acid substitutions [8,9].

*LGI1* is mainly expressed in neurons [1,10,11] and shows no similarity to known ion channels. The predicted structure of the LGI1 protein comprises a signal peptide, four leucine-rich repeats (LRRs) [12], and seven repeats named EPTP [13] or EAR [14] likely forming a beta-propeller structural domain [15]. Both LRR and beta-propeller domains mediate protein-protein interactions [15,16].

The LGI1 protein is secreted [10,17,18], and most ADLTE-causing *LGI1* mutations inhibit protein secretion [10,17,19–21], consistent with a loss-of-function effect of mutations. We recently reported the first disease-causing *LGI1* mutation (R407C) with no inhibitory effect on LGI1 secretion [22].

LGI1 has been implicated in various functions, some of which are mediated by interactions with two ADAM (A Disintegrin And Metalloprotease domain) receptors. LGI1 has been shown to bind to the postsynaptic receptor ADAM22 and this ligand-receptor complex participates in the control of synaptic strength at excitatory synapses [23]. It also binds to ADAM23 to stimulate neurite outgrowth both *in vitro* and *in vivo* [24] and may act as a trans-synaptic protein connecting the pre-synaptic ADAM23 with the post-synaptic ADAM22 receptors [25]. Though different in nature, each of these functions may potentially be related to epilepsy if impaired by mutations of *LGI1* that prevent or disturb interactions with ADAM22 and ADAM23 receptors.

Recent work has shown that serum LGI1 autoantibodies from patients with limbic encephalitis (LE), which is characterized by cognitive dysfunction and seizures [26, 27], prevent interaction of LGI1 with ADAM22 [28]. It has also been shown that some ADLTE-related mutations allowing secretion of LGI1 impair its binding to ADAM22 but not to ADAM23 [29]. In this paper we show that secretion-positive LGI1 mutations impair extracellular binding to both ADAM22 and ADAM23 receptors, providing further evidence for the importance of the LGI1-ADAM22/23 protein complex in the molecular mechanisms underlying ADLTE.

## Results

### Selection of study mutations

Previous 3D modelling of the LGI1 protein [30] predicted that some ADLTE-causing, nonsynonymous mutations in the C-terminal EPTP (beta-propeller) domain could allow secretion of the mutant protein and exert their pathogenic effects extracellularly, suggesting a distinction between correct protein folding and physiological function. In the present study, we used the 3D model of the LGI1 EPTP domain to envisage the possible effects of four disease-causing mutations, T380A, R407C, S473L and R474Q, on the structure or function of this protein region. The genetic features of these mutations are summarized in Table 1. The clinical features associated with three of them have been described [22,31,32], whereas the phenotype caused by the T380A mutation will be described elsewhere.

**Table 1:** Genetic features of study mutations.

Mutation	Mutated ADLTE family	Total number of patients	Occurrence in healthy controls^a^	Occurrence in ESP 5400 database^b^	Conservation of mutated residue	Polyphen-2 score^d^	SIFT score^e^	Reference
T380A	1	5	no	no	high^c^	0.937	0.13	[42]
S473L	2	14	no	no	high	1.000	0.09	[27; 28]
R474Q	1	3	no	no	high	1.000	0.00	[28]
R407C	1	3	no	no	high	0.998	0.00	[22]

^a^ 50–150 geographically matched healthy controls were screened for each mutation

^b^ the GO Exome Sequencing Project of the National Heart, Lung, and Blood Institute (http://evs.gs.washington.edu/EVS)

^c^ conservation in all vertebrate species

^d^ 0.000–0.850, benign; 0.851–0.950, possibly damaging; 0.951–1.000, probably damaging

^e^ 0.00–0.05, not tolerated; 0.06–0.20, potentially not tolerated; 0.21–1.00, tolerated.

### *In silico* prediction of the effects of study mutations

**Fig 1:**
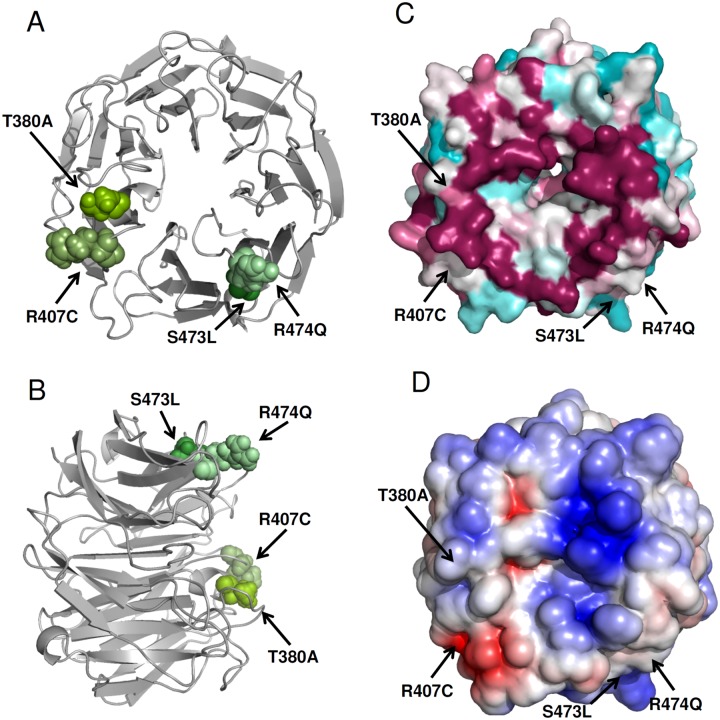
Mapping of the four mutated residues resulting in secreted LGI1 mutant proteins on the human EPTP beta-propeller model. In A and B the beta-propeller model is represented as a cartoon in front and in lateral view to the top surface, respectively. Green spheres indicate atoms of mutated residue side chains resulting in secreted LGI1 mutant proteins. C: conserved surface of the human EPTP beta-propeller calculated with Consurf colouring from unconserved (cyan) to strictly conserved (magenta). The conservation degree was calculated between human LGI proteins and their orthologs (Mus musculus, Rattus norvegicus, Bos taurus, Xenopus tropicalis, and Danio rerio). D: electrostatic surface of the EPTP beta-propeller (negative charge in red and positive charge in blue).

Fig 1 shows the LGI1 EPTP domain with the T380A, S473L, R474Q, and R407C mutated residue positions highlighted and additional information on conservation and electrostatics on the protein surface. All four mutated residues lie on the surface of the beta-propeller domain within or very close to a ring of conserved residues located on the top surface, which has been predicted to be crucial for the protein interactions mediated by the EPTP domain [30]. Amino acid substitutions perturbing the organization of this area may affect LGI1 function by altering the interaction surface selectively while leaving the protein fold virtually unaffected. This situation is remarkably different from that predicted for mutations suppressing LGI1 secretion, which likely alter the folding of either LRR or EPTP domain [see ref. 30], and may provide valuable clues into LGI1 function arising through protein-protein interactions.

### Study mutations do not inhibit LGI1 secretion

**Fig 2:**
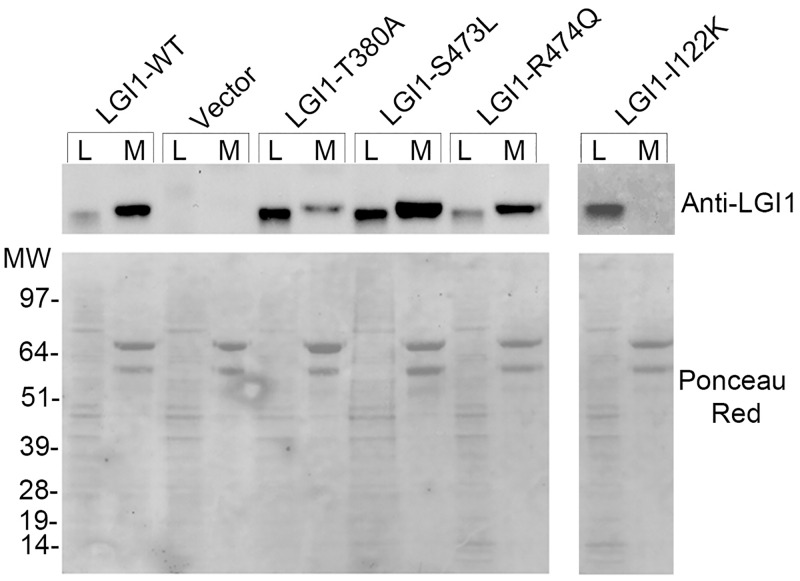
Secretion test of wild type and mutated LGI1. Western blot of cell lysates (L) and concentrated media (M) of HEK293T cells transfected with (left panel) LGI1 wild type or sec+ mutant, c.1138A>G (T380A), c.1418C>T (S473L), and c.1421G>A (R474Q), expression constructs, or with (right panel) the LGI1 sec- mutant, c.365T>A (I122K), construct. Bands were revealed with an anti-LGI1 antibody. Ponceau S staining of the blots are shown below. Vector, empty vector; MW, molecular weight markers.

The inability of the R407C substitution to suppress LGI1 secretion was demonstrated previously [22]. To ascertain the consequences of the *LGI1* c.1138A>G (T380A), c.1418C>T (S473L), and c.1421G>A (R474Q) mutations on secretion of LGI1, we transfected expression constructs containing the wild type and mutated *LGI1* cDNAs into human embryonic kidney 293T (HEK293T) cells and analyzed cell lysates and concentrated conditioned media by western blot using an anti-LGI1 antibody. All three mutant proteins as well as the wild type control were detected in the conditioned media of transfected cells and, though in variable amounts, in the cell lysates (Fig 2). We consistently observed in three independent experiments a low amount of LGI1-T380A in the medium (31% of the secreted wild type protein; S1 Fig), suggesting that secretion of this mutant protein was partially hampered (see Discussion). In contrast, the LGI1 mutant protein carrying the pathogenic I122K substitution [21] was not secreted from transfected cells (Fig 2), as previously shown for many other ADLTE-causing single amino acid substitutions in both LRR and EPTP regions [10,17,19–21]. Thus, according to this secretion assay, the S473L and R474Q mutations do not inhibit secretion of LGI1, as it was the case for R407C, whereas T380A allows secretion of part of the mutated protein. For this reason all four study mutations are collectively named here sec+ mutations, whereas mutations inhibiting LGI1 secretion are termed sec- mutations.

### LGI1 mutant proteins have no effects on secretion of co-expressed wild type LGI1

**Fig 3:**
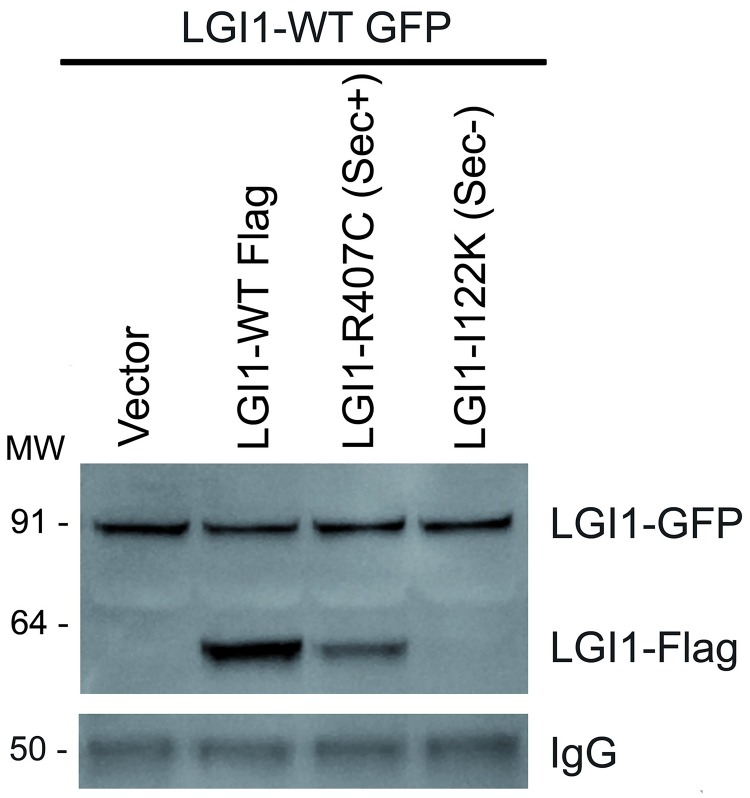
Secretion of wild type LGI1 co-expressed with LGI1 mutant proteins. Western blot of concentrated media of HEK293T cells co-transfected with GFP-tagged wild type (wt) LGI1 and one of the indicated Flag-tagged LGI1 constructs: wt, R407C (sec+), or I122K (sec-). Bands were revealed with an anti-LGI1 antibody. IgG gel loading control added to cell culture medium prior concentration is shown below. Vector, empty vector; MW, molecular weight markers.

In ADLTE patients carrying *LGI1* heterozygous mutations, allelic wild type and mutant proteins are synthesized in the same neuronal cells. To test whether mutant proteins could perturb secretion of co-expressed wild type LGI1, we co-transfected HEK293T cells with wild type LGI1 tagged with green fluorescent protein (GFP) and either LGI1-R407C (sec+) or LGI1-I122K (sec-) flagged constructs and determined the amounts of secreted LGI1-GFP by western blot. Fig 3 shows that neither the sec+ nor the sec- mutant proteins altered the amount of secreted LGI1-GFP. Repeated experiments yielded consistent results, indicating that both types of mutant proteins do not interfere with the secretion process of wild type LGI1 synthesized in the same cells.

### Study mutations affect interactions with ADAM22/23: Immunofluorescence

**Fig 4:**
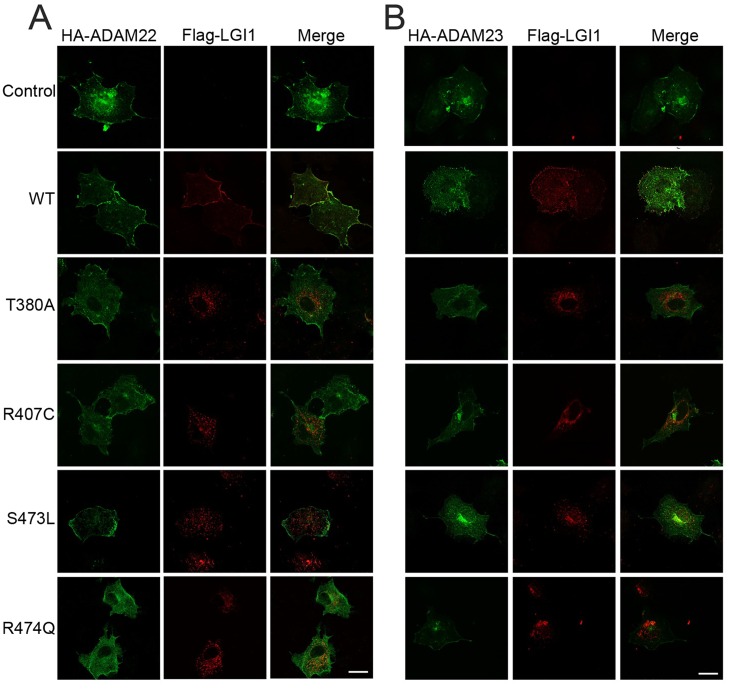
Interaction between secreted LGI1 and ADAM22 (panel A) or ADAM23 (panel B) on the cell membrane. COS7 cells were transiently co-transfected with wild type or mutated LGI1-Flag and HA-tagged ADAM22 or ADAM23 expression constructs. Thirty-six hours after transfection, the LGI1 proteins were labelled with anti-Flag antibody (red), then cells were permeabilized, and ADAM proteins were stained with anti-HA antibody (green). Each panel displays representative confocal microscopy images, where wild type LGIi1 co-localized with ADAM receptors on the cell surface, whereas mutant LGI1 proteins failed to interact with ADAM receptors. Scale bars, 10 μm.

To investigate whether the four sec+ mutations affected the interaction of LGI1 with ADAM22/23 receptors, we overexpressed the wild type and mutant *LGI1*-Flag cDNAs together with HA-fused *ADAM22* or *ADAM23* constructs in COS7 cells. Thirty-six hours after co-transfection, cells were stained with anti-Flag and anti-HA antibodies and double-immunofluorescence analysis was carried out using confocal imaging. As exemplified in Fig 4, the wild type LGI1 protein, when co-expressed with either ADAM22 or ADAM23, mostly co-localized with either receptor on the cell membrane, whereas LGI1 mutant molecules failed to interact or interacted poorly with ADAM22 and ADAM23 receptors. This was consistently observed in three different experiments. Overall, the percentage of sec+ mutant proteins bound to either ADAM receptor on the cell membrane (0–33%) was significantly lower than that of wild-type LGI1 (76–88%; Chi-square test, p < 0.0001) (Table 2).

**Table 2:** Percentage of cells with LGI1 (wild type or mutated) bound to either ADAM22 or ADAM23 receptor on the cell membrane.

		LGI1				
		WT	T380A	R407C	S473L	R474
**ADAM22**	**Total cells**	115	81	90	97	86
	**Membrane-bound LGI1**	87 (76%)	0 (0%)	32 (36%)	3 (3%)	11 (13%)
**ADAM23**	**Total cells**	107	78	72	63	62
	**Membrane-bound LGI1**	94 (88%)	0 (0%)	21 (29%)	10 (16%)	14 (23%)

All frequency differences between wild-type (WT) and mutated LGI1 are statistically significant (Chi-square test, p<0.0001).

### Secreted LGI1 binds to the extracellular domain of ADAM22

Blood sera from patients with LE containing high titre of LGI1 autoantibodies [26,27] have been shown to neutralize the LGI1-ADAM22 interaction [28]. To show that co-expressed LGI1 and ADAM22 interact on the cell surface, we co-transfected wild type LGI1-Flag and HA-ADAM22 cDNAs into COS7 cells that were cultured in conditioned medium containing serum from an LE patient. We consistently found in three different experiments that increasing concentrations of LE serum in the conditioned media progressively reduced binding of LGI1 to membrane-bound ADAM22 (total cell counts in Table 3). This result demonstrates that secreted LGI1 binds to the extracellular domain of ADAM22, and that this interaction is disturbed by extracellular LGI1 autoantibodies.

**Table 3:** Proportion of cells with LGI1-ADAM22 complex on the outer membrane surface in the presence or absence of LE patient serum.

LE serum (%)	Total cells	Cells with membrane-bound LGI1
0	115	87 (76%)
2.5	273	102 (37%)
5	254	98 (38%)
10	236	47 (20%)

A statistically significant difference (P<0.0001) was observed for all LE serum concentrations versus control (0% serum)

### Study mutations affect interactions with ADAM22/23: Co-immunoprecipitation

**Fig 5:**
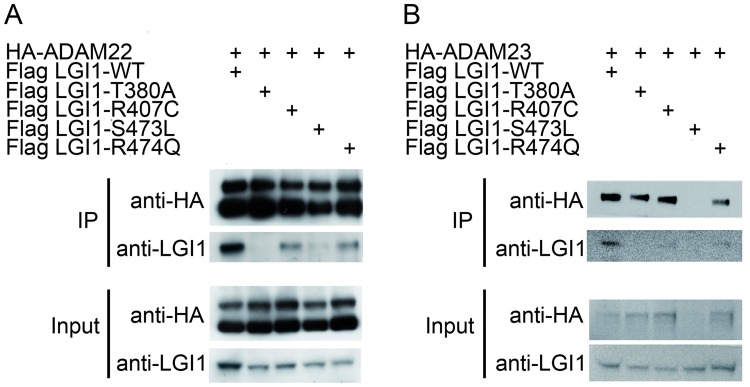
LGI1 mutations impair interaction with ADAM22 and ADAM23. HEK293T cells were transiently co-transfected with (A) HA-ADAM22 or (B) HA-ADAM23 and LGI1 wild-type or mutants. Inputs (6.5% of total proteins) and HA-immunoprecipitations were separated in SDS-PAGE, transferred and immunoblotted using the indicated antibody. Co-immunoprecipitations showed that wild type LGI1 interacts with HA-ADAM22 (A) and HA-ADAM23 (B), while the interaction is abolished in the presence of T380A mutant. LGI1 mutants R407C, S473L and R474Q displayed a reduced interaction compared to the wild-type. Each of these experiments was performed twice with similar results. In the experiment shown in panel B, HA-ADAM23 failed to transfect HEK293T cells together with LGI1-S473L.

To independently assess whether LGI1 mutations affect binding to ADAM22 and ADAM23, we performed co-immunoprecipitation assays from HEK293T cells overexpressing HA-tagged ADAM22 or HA-ADAM23 and LGI1-Flag. As shown in Fig 5A, HA-ADAM22, which is present in both immature (110 kDa) and mature (90 kDa) forms as previously observed (PMID:20156119), efficiently co-precipitated wild type LGI1. Instead, all LGI1 sec+ mutations affected LGI1-ADAM22 interactions: the T380A mutation completely disrupted the interaction, the S473L mutation almost abolished it, whereas R407C and R474Q reduced the affinity for ADAM22. We also assessed the effect of sec+ mutations on LGI1-ADAM23 interaction using co-immunoprecipitation. As shown in Fig 5B, only wild type LGI1 was clearly immunoprecipitated by ADAM23, consistent with the results obtained with ADAM22, while pathological LGI1 mutations displayed reduced affinity for ADAM23. We obtained consistent results from two experiments. In both cases expression of HA-ADAM23 was found to be lower than that of HA-ADAM22, resulting in a lower efficiency of LGI1 immunoprecipitation.

## Discussion

We show in this paper that several ADLTE-causing *LGI1* mutations affecting amino acids of the C-terminal EPTP domain allow protein secretion and reduce affinity of secreted LGI1 for the ADAM22 and ADAM23 neuronal receptors. We also show that secreted LGI1 binds to ADAM22 on the cell surface, which was assumed but not demonstrated in previous works, and that secretion of wild type LGI1 is not influenced by LGI1 mutant proteins co-expressed in the same cells, ruling out any dominant-negative effect of both sec- and sec+ mutant proteins on trafficking and secretion of wild type LGI1.

Inhibition of secretion has long been the sole demonstrated mechanism by which *LGI1* mutations cause loss of protein function [8]. Recently we and others reported ADLTE-causative *LGI1* mutations that do not inhibit protein secretion [22, 29], indicating that LGI1 mutations can be either secretion-defective (sec-) or secretion-competent (sec+). Both types of mutations have loss-of-function effects, but sec+ mutations appear to exert their effect extracellularly by decreasing molecular affinity for both ADAM22 and ADAM23. Overall, out of the 22 *LGI1* missense mutations tested for secretion [10,17,19–22,29,33, and present work], four (18%) are sec+, all giving rise to an amino acid substitution in the EPTP domain. The presence of a comparatively large fraction of ADLTE-causing mutations that do not affect LGI1 secretion suggests that they allow the protein fold to be maintained while disrupting LGI1 interactions with other proteins. The surface residues replaced by sec+ mutations might either be directly responsible for the LGI1-ADAM22/23 interactions, or perturb, when mutated, the capability of nearby residues to interact with critical amino acids on the surface of ADAM22 and ADAM23. Particularly, any mutation disturbing the conserved residues forming a ring on the EPTP top surface (see Fig 1) might weaken the interaction or even abolish it entirely.

In a recent work, Yakoi et al. [29] showed that the sec+ mutation S473L decreased binding of LGI1 to ADAM22, but interaction with ADAM23 remained unchanged. They concluded that the pathogenic mechanisms underlying ADLTE in the case of sec+ mutations might result mostly, if not completely, from a dysfunction of the LGI1-ADAM22 complex. In contrast, we found that sec+ mutant proteins exhibit reduced binding affinity to both ADAM22 and ADAM23. This discrepancy may be explained by the different assays employed. Compared with the tandem-affinity purification of LGI1 from brain tissue of transgenic mice expressing mutant proteins used by Yakoi and co-workers, our optimized cell-based assays (36 h incubation after co-transfection; see Methods) may be more sensitive in detecting the decreased binding ability of sec+ mutations. Thus, our results suggest that both ADAM receptors likely play a role in the pathogenesis of ADLTE through their interactions with LGI1.

Genetic disorders that are caused by defects in a ligand for a particular receptor are frequently mirrored by disorders in which that receptor is deficient. However, a causative role for ADAM22 and ADAM23 in ADLTE has been questioned in genetic studies of families without *LGI1* mutations: direct sequencing of *ADAM22* exons revealed no disease-causing mutations [34,35], and linkage analysis with microsatellite markers within or near the *ADAM23* gene failed to reveal any significant linkage peak [36]. The absence of causative mutations in *ADAM22* and *ADAM23*, however, is not in contrast with their involvement in the molecular pathway underlying ADLTE, and this apparent contradiction can be explained in different ways. It is possible that mutations in these genes have not been detected in the limited set of ADLTE families tested because they occur at low frequency, a hypothesis supported by recent studies suggesting a relatively high genetic heterogeneity in ADLTE families free of *LGI1* mutations [7]. Alternatively, *LGI1*-associated ADLTE may reflect a partial loss of function of the LGI1 ligand at both receptor proteins ADAM22 and ADAM23, whereas heterozygous mutations in only one of these receptors may not be sufficient to cause the syndrome. The recent identification of *ADAM22* compound heterozygous mutations in a patient with severe encephalopathy and epilepsy, who inherited the mutations by healthy parents, supports the hypothesis that *ADAM22* may primarily be a recessive disease gene [37]. Also, protein models suggesting that LGI1 is able to interact with ADAM22 and ADAM23 simultaneously [25,30] provide support to this interpretation, which might also apply to other as yet unknown proteins interacting with LGI1. Further support to the importance in epileptic disorders of the ligand-receptor interaction between LGI1 and ADAM22/23 comes from a recent study showing that these interactions are specifically impaired by LGI1 autoantibodies from the sera of patients with limbic encephalitis, which is characterized by amnesia and seizures [28]. Reduced binding of LGI1 to ADAM22/23 may therefore be a pathogenic mechanism for both genetically inherited and acquired epilepsy disorders.

Recent work has shown that haploinsufficiency caused by sec- mutations does not result from lack of protein secretion but from intracellular degradation of misfolded mutant proteins by the endoplasmic reticulum protein quality-control mechanisms [29]. This is in agreement with the predictions of our 3D model for sec- mutations, which likely destabilize protein folding [30], and also provides a possible explanation for the low amount of secreted LGI1-T380A we observed. This mutant protein was found virtually absent in the cell culture medium in a previous work [29], in which the T380A substitution was therefore regarded as a sec- mutation. Instead, we found a low but consistently detectable amount of LGI1-T380A in the cell medium (S1 Fig). Although we regarded it as a sec+ mutation for simplicity, the T380A substitution is not readily classifiable because only a relatively low proportion of the mutant protein appears to be secreted, whereas the rest of the mutant molecules might be degraded in the endoplasmic reticulum, possibly due to a mild alteration of the EPTP domain folding resulting from this mutation. In any case, the secreted LGI1-T380A protein failed to bind to both ADAM receptors under our experimental conditions, suggesting a loss-of-function effect of this genetic defect. Despite its possible mixed effects on protein stability and secretion, this mutation leads to the same clinical phenotype caused by *LGI1* sec+ as well as sec- mutations.

The discovery of sec+ mutations provides new opportunities for investigating LGI1 functions and understanding their relevance to ADLTE. For example, the study of sec+ mutant proteins in in vitro cell systems may help clarify whether some functions attributed to LGI1, such as control of dendrite growth and cell adhesion, are related to ADLTE; also, animal models incorporating these types of mutations may provide new insights into the role of LGI1 in processes such a synaptic maturation and transmission. These studies will improve our comprehension of the pathogenic mechanisms of ADLTE as a paradigm of non-ion channel idiopathic epilepsy. Moreover, new therapeutic strategies that make use of chemical correctors to restore protein folding might be effective with LGI1 sec- mutations [29] but not with sec+ mutations, which have little or no effect on protein folding. Therefore, a correct classification of LGI1 mutations based on their effects of protein folding and secretion might have in the future important therapeutic implications.

## Materials and Methods

### *In silico* analysis

Both the LGI1 beta-propeller structure model as recently published [30] and the crystal structure of ADAM22, PDB identifier 3G5C [38], were visualized using PyMOL (DeLano Scientific, URL: http://pymol.sourceforge.net/). The degree of conservation on the protein surface was mapped with ConSurf [39], using all known LGI1 through LGI4 homologs and the ADAM22 orthologs from OMAbrowser [40] respectively. The electrostatic surface was calculated with BLUUES [41] and mapped on the protein structures. The pathogenicity of four LGI1 mutations has been predicted also using the two widely used computational methods, SIFT (http://sift.bii.a-star.edu.sg/) and Polyphen-2 (http://genetics.bwh.harvard.edu/pph2/). The SIFT score indicates the degree of conservation derived from a sequence alignment of closely related proteins. Polyphen-2 is based on both sequence and structural information. It uses the PSIC (Position-Specific Independent Count) software to calculate a profile matrix for each position in the sequence alignment. The difference between profile scores of allelic variants indicates the probability of the substitution to be observed in the protein family at that position.

### Antibodies

Rabbit anti-LGI1 antibodies were from Abcam (Cambridge, UK; catalog No. ab30868); mouse anti-HA.11, clone No. 16B12, from Covance (Princeton, NJ, USA); rabbit anti-Flag from Sigma Aldrich (St. Louis, MO, USA; catalog No. F7425). Secondary antibodies Alexa-Fluor 488-conjugated goat anti-mouse IgG were from Life Technology (Grand Island, NY, USA; catalog No. A11001); and Cy3-conjugated goat anti-rabbit IgG from Jackson ImmunoResearch Laboratories (West Grove, PA, USA; catalog No. 111-165-003). Horseradish peroxidase-conjugated mouse-specific IgG, catalog No. P0260, and horseradish peroxidase-conjugated rabbit-specific IgG, catalog No. P0448, were from DakoCytomation (Denmark A/S).

### LGI1 secretion assay

Cell-based LGI1 secretion assay was performed as described in detail previously [18]. Briefly, expression constructs containing the *LGI1* wild type or mutant cDNAs were transfected into HEK293T cells using Lipofectamine 2000 (Life Technology), following the manufacturer instructions. Twenty-four hours after transfection, cells were washed twice and then re-fed with serum-free medium Opti-MEM (Life Technology). After 16–20 hours, the medium was collected and centrifuged to pellet cell debris, and the supernatant was concentrated about 20x using Centricon YM30 concentrators (Merk-Millipore, Billerica, MA, USA). Cells were lysed in Triton lysis buffer (25 mM Tris pH 7.4, 150 mM NaCl, 1% (vol/vol) Triton, 10% (vol/vol) Glycerol, 1mM EDTA) supplemented with proteases and phosphatase inhibitors.

Cell proteins (14 μg/lane) and concentrated media were separated on 12% NuPAGE (Life Technology) and then electroblotted onto nitrocellulose membrane. The integrity of the Western blot was analysed by Red Ponceau staining. Destained membranes were blocked with 10% (w/v) skimmed milk in tris-buffered saline (TBS) for 1 hr and then incubated with primary antibody in TBS containing 2% (vol/vol) skimmed milk for 2 hrs at room temperature. Proteins immunostained with anti-LGI1 antibody were detected with a horseradish peroxidase-labelled secondary antibody and enhanced chemiluminescence reagent and visualized by autoradiography.

To examine the effect of LGI1 mutant proteins on secretion of wild type LGI1, HEK293T cells were co-transfected with wild type LGI1-GFP and a Flag-containing LGI1 mutant, either c.1219C>T (R407C) or c.365C>A (I122K), constructs (1.5 ug each). Following cell incubation, serum-free conditioned medium was concentrated and analyzed by western blot as described above. As gel loading control, a fixed quantity of IgG (14 ng) was added to 6 ml cell medium before concentration (60x) so as to load approximately 2 ng of IgG in each gel lane. The IgG were detected with a horseradish peroxidase-labelled secondary antibody. This experiment was repeated three times to confirm reliability of results.

### COS7 cell co-transfection and immunofluorescence

COS7 cells were seeded on sterile glass coverslips and co-transfected with wild-type or mutant *LGI1*-Flag and HA-tagged *ADAM22* or *ADAM23* cDNAs (3 ug of total DNA). Thirty-six hours after transfection, cells were fixed with 4% paraformaldehyde (PFA) at room temperature for 10 min and blocked with PBS containing 2 mg/ml bovine serum albumin (BSA) (Sigma-Aldrich) for 40 min. Fixed cells were stained with rabbit anti-Flag antibody (1:300), followed by Cy3-conjugated anti-rabbit secondary antibody (1.300). Then, the cells were permeabilized with 0.1% Triton X-100 for 10 min, blocked with PBS containing 2mg/ml BSA and stained with anti-HA monoclonal antibody (1:500), followed by Alexa-Fluor 488-conjugated anti-mouse secondary antibody (1:500). All the antibodies were diluted in 1% BSA/PBS; this was followed by washes with PBS. Finally, they were fixed with mounting medium containing DAPI (Vectashield; Vector Laboratories). Two coverslips were made for every transfection experiment, and twenty random fields were taken (magnification 400X). For every field the number of co-immunostained cells (LGI1 and ADAM 22 or ADAM 23) was counted and the percentage of cells with both signals on the cell surface was estimated. In total, three independent experiments were performed. For cell counting, slides were analysed using a Leica-DM 5000B Epifluorescence microscope. Confocal images were acquired with a Radiance 2000 confocal microscope (BioRad).

The GraphPad software program (http://www.graphpad.com/quickcalcs/) was used to calculate Chi-square and the two-tailed P-value to compare the frequency of membrane-bound LGI1 proteins between cells carrying the wild type LGI1 protein and cells expressing LGI1 mutated proteins.

Blood serum from a patient with LGI1-positive autoimmune LE was used to investigate weather interaction of LGI1 with ADAM22 occurred on the outer surface of the cell membrane. COS7 cells were seeded on sterile glass coverslips and co-transfected with wild type LGI1-Flag and HA-tagged ADAM22 cDNAs as described above. Twenty hours after transfection the medium was replaced with serum-free medium additioned with 2,5%, 5% and 10% LE patient serum. After twenty-eight hours cells were processed and immunofluorescence analysis was performed as described above. Statistical analysis was performed as described above.

### Co-immunoprecipitation

LGI1-Flag wild-type or clinical mutants were co-transfected with HA-ADAM22 or HA-ADAM23 into HEK293T cells seeded on a six wells plate. Thirty-six hours after transfection, cells were lysed in 200 μl of lysis buffer (50 mM Tris-HCl, pH 7.5, 1 mM EDTA, 1 mM sodium orthovanadate, 10 mM sodium β-glycerophosphate, 5 mM sodium pyrophosphate, 0.27 M sucrose and 1% (w/v) Tween 20 in the presence of lx protease inhibitor cocktail (Sigma Aldrich) and incubated on ice for 20 minutes. Lysates were subsequently clarified by centrifugation at 14000 g for 20 minutes and 0.5 mg of total proteins in 500 μl of volume were incubated with 0.5 μg of mouse monoclonal anti-HA antibody (Roche). After 2 hours of incubation at 4°C with gentle rocking, 20 μl of Protein G sepharose (GE Healthcare Life Sciences) were supplemented and incubated 1 hour at 4°C. After 5 washes in washing buffer (50 mM Tris/HCl, pH 7.5, 1 mM EDTA, 1 mM sodium orthovanadate, 10 mM sodium β-glycerophosphate, 5 mM sodium pyrophosphate, 0.27 M sucrose, 1% (v/v) tween 20 and 250 mM NaCl), proteins were eluted in 2x sample buffer (Invitrogen), separated in NuPAGE 4–12% (Life Technology) and electro-blotted on polyvinylidene fluoride membrane (PVDF) (Merk-Millipore). PVDF membranes were saturated with 10% skimmed milk in TBS supplemented with 0.05% tween 20 and proteins detected using mouse primary anti-HA (1:1000) and rabbit anti-LGI1 (1:1000) antibodies and secondary antibodies conjugated with horseradish peroxidase mouse- specific IgG and rabbit-specific IgG (both diluted 1:2000).

## Supporting Information

S1 Fig Quantification of secretion levels of three LGI1 mutant proteins. Protein amounts detected in cell media by western blot in three independent experiments are expressed in percentage as compared to wild type LGI1.
(DOC)
