# Supplementary material for: Secretion-Positive LGI1 Mutations Linked to Lateral Temporal Epilepsy Impair Binding to ADAM22 and ADAM23 Receptors
Source: PLoS Genet. 2016 Oct 19;12(10):e1006376. doi: 10.1371/journal.pgen.1006376 (PMC5070869; doi:10.1371/journal.pgen.1006376)
Supplement: S1 Fig — Protein amounts detected in cell media by western blot in three independent experiments are expressed in percentage as compared to wild type LGI1. (DOC) [file pgen.1006376.s001.doc]

**Supporting Information**

Secretion-positive LGI1 Mutations Linked to Lateral Temporal Epilepsy Impair Binding to ADAM22 and ADAM23 Receptors

Emanuela Dazzo, Emanuela Leonardi, Elisa Belluzzi, Sandro Malacrida, Libero Vitiello, Elisa Greggio, Silvio C.E. Tosatto, Carlo Nobile

Figure S1


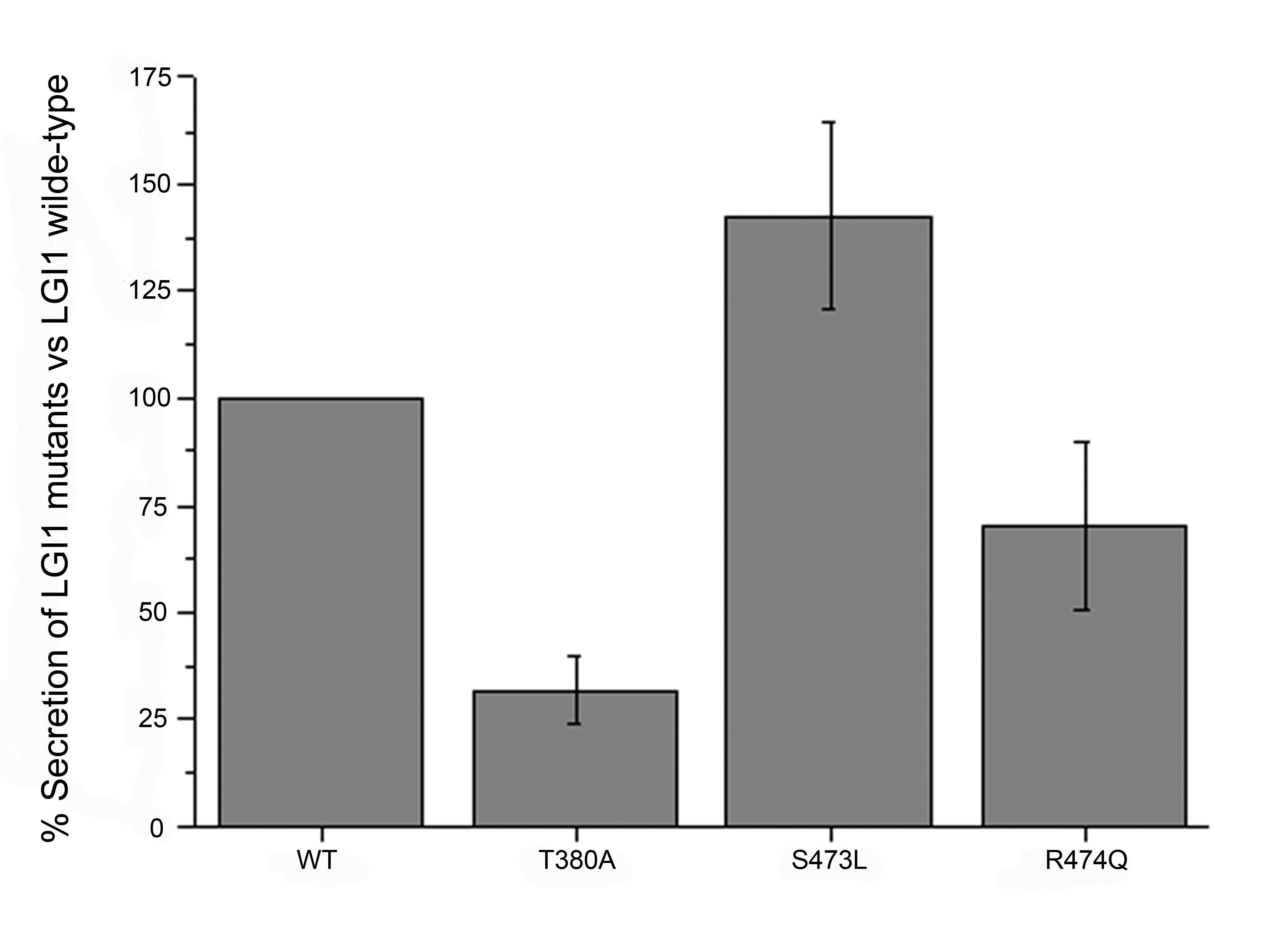


**Figure S1.** Quantification of secretion levels of three LGI1 mutant proteins. Protein amounts detected in cell media by western blot in three independent experiments are expressed in percentage as compared to wild type LGI1.
